# Supplementary material for: Effect of N-Acetylcysteine in Combination with Antibiotics on the Biofilms of Three Cystic Fibrosis Pathogens of Emerging Importance
Source: Antibiotics (Basel). 2021 Sep 27;10(10):1176. doi: 10.3390/antibiotics10101176 (PMC8532722; doi:10.3390/antibiotics10101176)
Supplement: Supplementary file 1 [file antibiotics-10-01176-s001.zip › antibiotics-1378188-supplementary.pdf]

Supplementary Materials

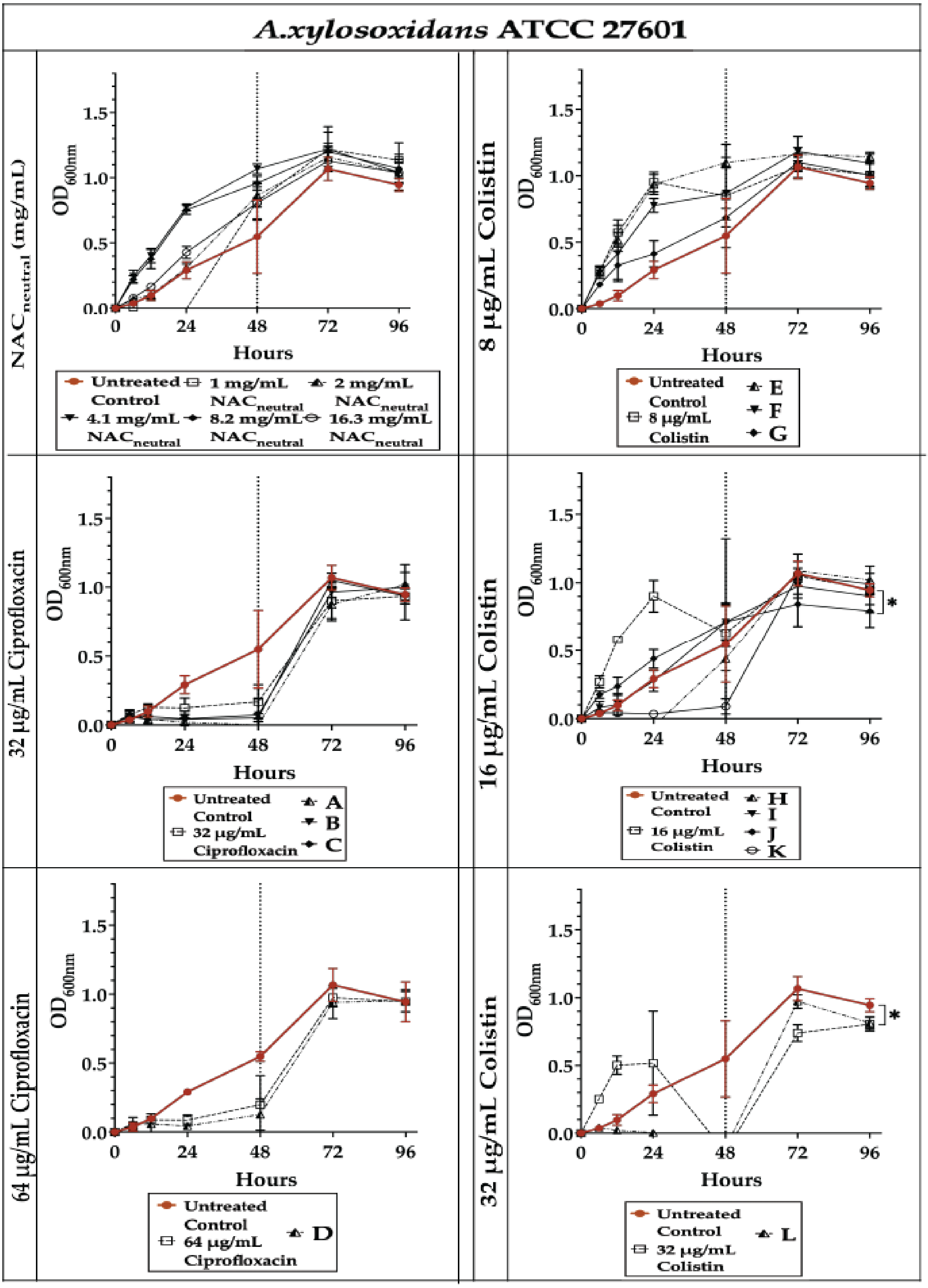

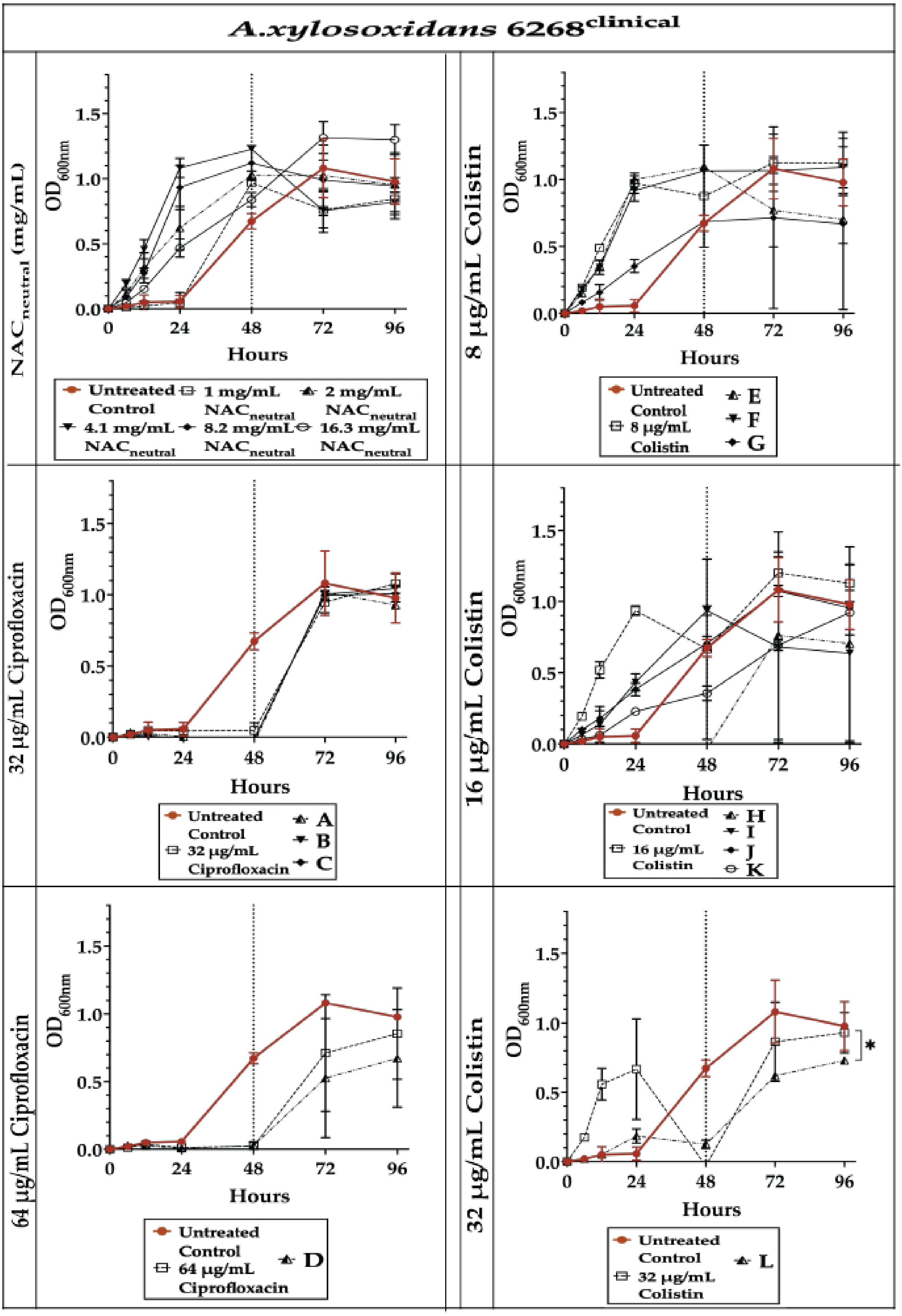

A.xylosoxidans 6268clinical
NACneutral (mg/mL)
8 µg/mL Colistin
32 µg/mL Ciprofloxacin
16 µg/mL Colistin
64 µg/mL Ciprofloxacin
32 µg/mL Colistin
OD600nm
Hours
0.0
0.5
1.0
1.5
0
24
48
72
96
Untreated Control
1 mg/mL NACneutral
2 mg/mL NACneutral
4.1 mg/mL NACneutral
8.2 mg/mL NACneutral
16.3 mg/mL NACneutral
8 µg/mL Colistin
E
F
G
32 µg/mL Ciprofloxacin
A
B
C
16 µg/mL Colistin
H
I
J
K
64 µg/mL Ciprofloxacin
D
32 µg/mL Colistin
L
*

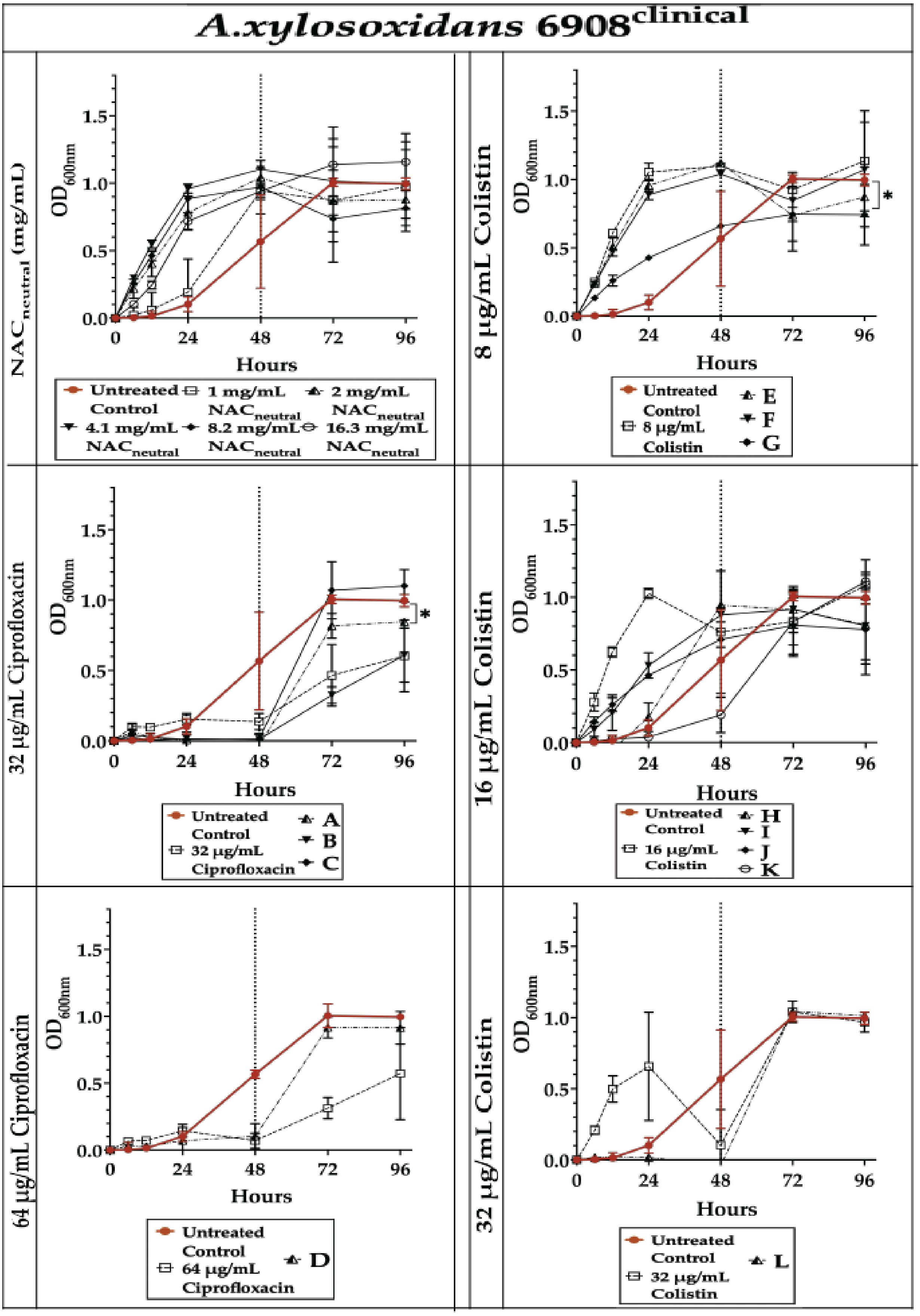

A.xylosoxidans 6908clinical
NAC neutral (mg/mL)
8 μg/mL Colistin
32 μg/mL Ciprofloxacin
16 μg/mL Colistin
64 μg/mL Ciprofloxacin
32 μg/mL Colistin
OD600nm
Hours
Untreated Control
1 mg/mL NACneutral
2 mg/mL NACneutral
4.1 mg/mL NACneutral
8.2 mg/mL NACneutral
16.3 mg/mL NACneutral
8 μg/mL Colistin
E
F
G
32 μg/mL Ciprofloxacin
A
B
C
16 μg/mL Colistin
H
I
J
K
64 μg/mL Ciprofloxacin
D
32 μg/mL Colistin
L
*

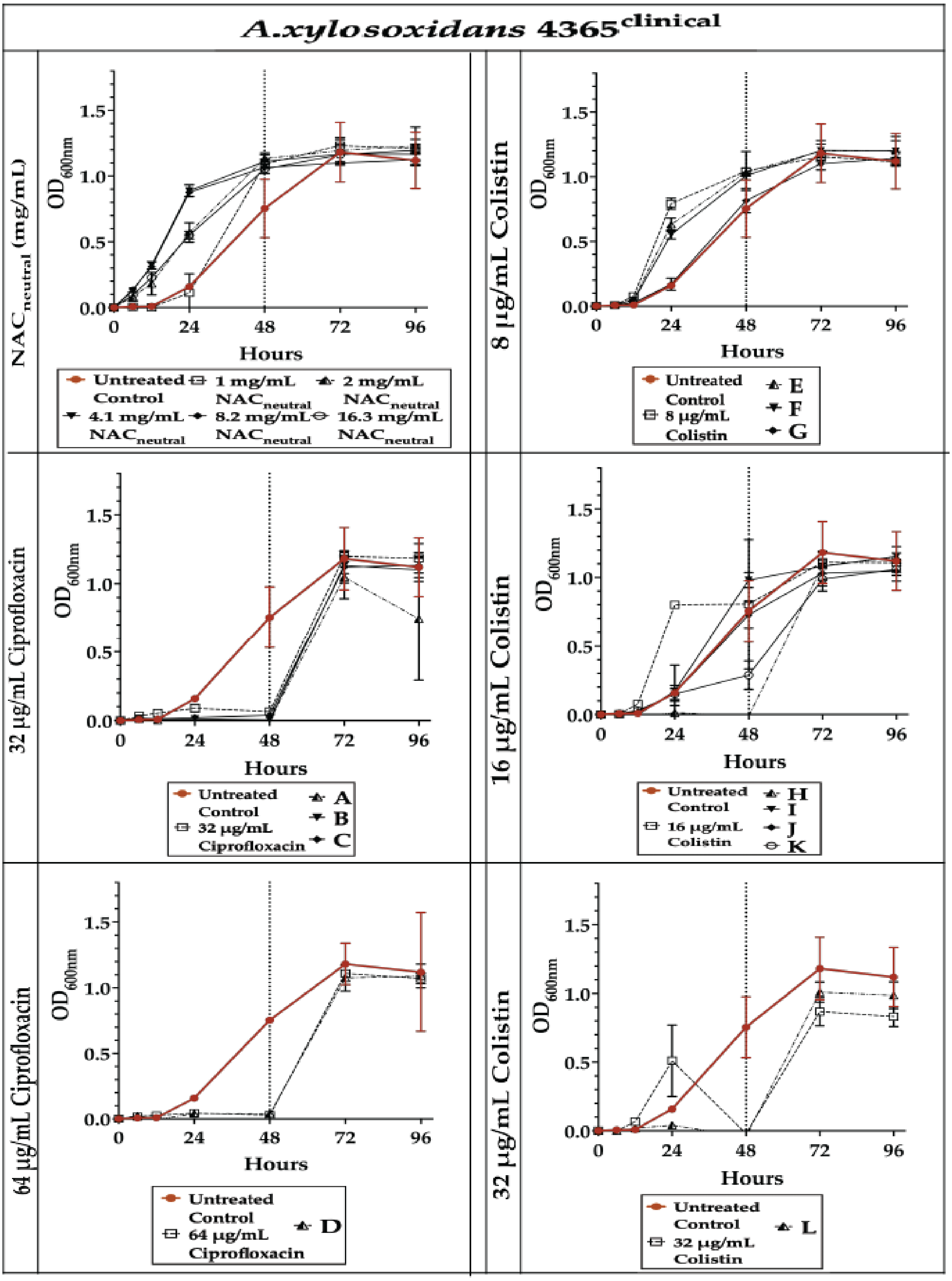

Figure S1. Bacteriostatic effect of NACneutral and antibiotic combinations on *A. xylosoxidans* growth. The vertical dotted line running through the 48 h timepoint signifies the point at which treatment was removed, washed and replaced with fresh tryptone soya broth (TSB). Statistical analyses were performed using unpaired Student's t-tests (with Welch's correction) against final timepoint at 96 h. Significance cut-offs are as follows $P \leq 0.05$ (*). Data represent an average of n = 3 biological replicates.

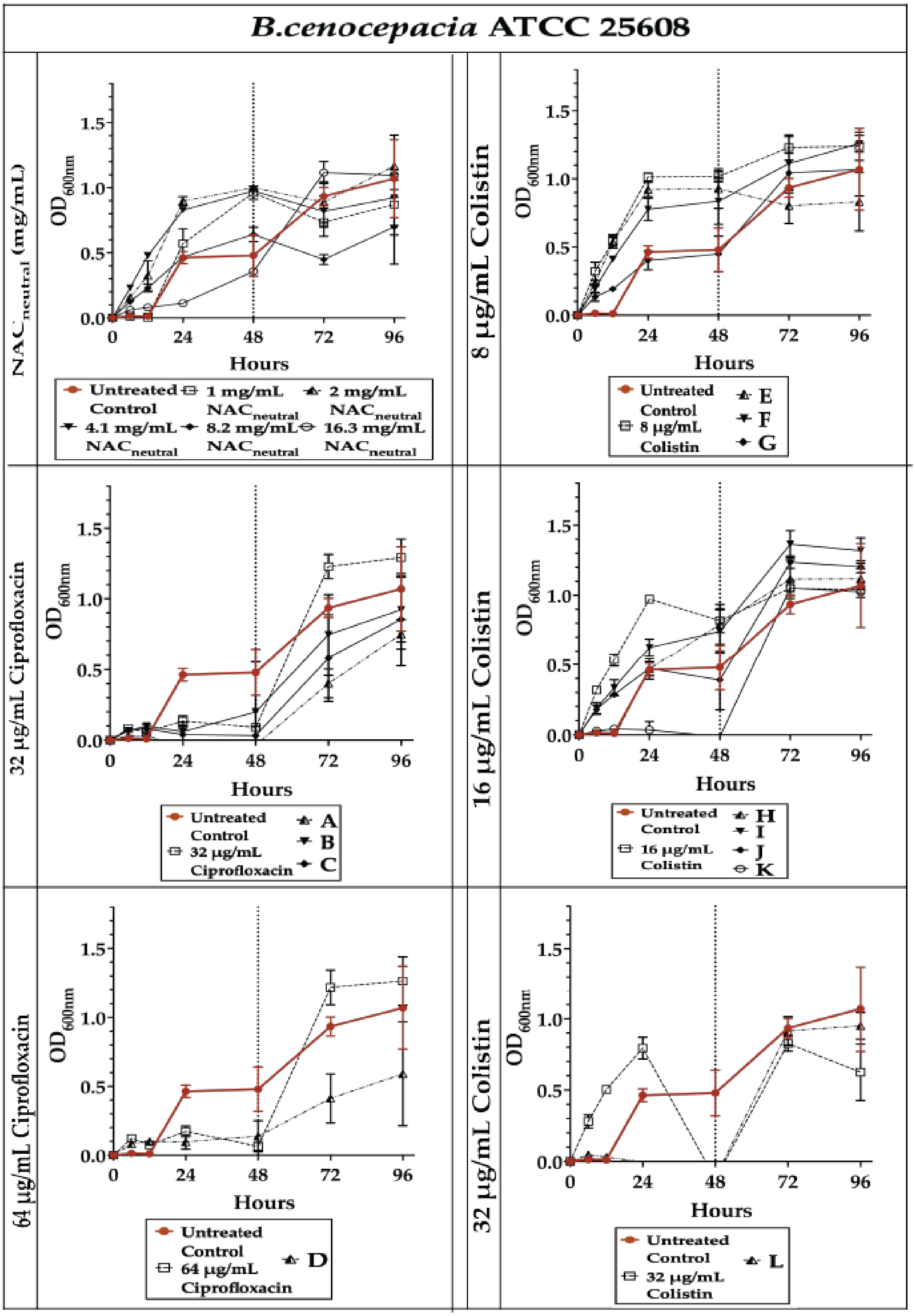

B.cenocepacia ATCC 25608
NAC$_{neutral}$ (mg/mL)
OD$_{600nm}$
Hours
Untreated Control
1 mg/mL NAC$_{neutral}$
2 mg/mL NAC$_{neutral}$
4.1 mg/mL NAC$_{neutral}$
8.2 mg/mL NAC$_{neutral}$
16.3 mg/mL NAC$_{neutral}$
8 μg/mL Colistin
Untreated Control
8 μg/mL Colistin
E
F
G
32 μg/mL Ciprofloxacin
Untreated Control
32 μg/mL Ciprofloxacin
A
B
C
16 μg/mL Colistin
Untreated Control
16 μg/mL Colistin
H
I
J
K
64 μg/mL Ciprofloxacin
Untreated Control
64 μg/mL Ciprofloxacin
D
32 μg/mL Colistin
Untreated Control
32 μg/mL Colistin
L

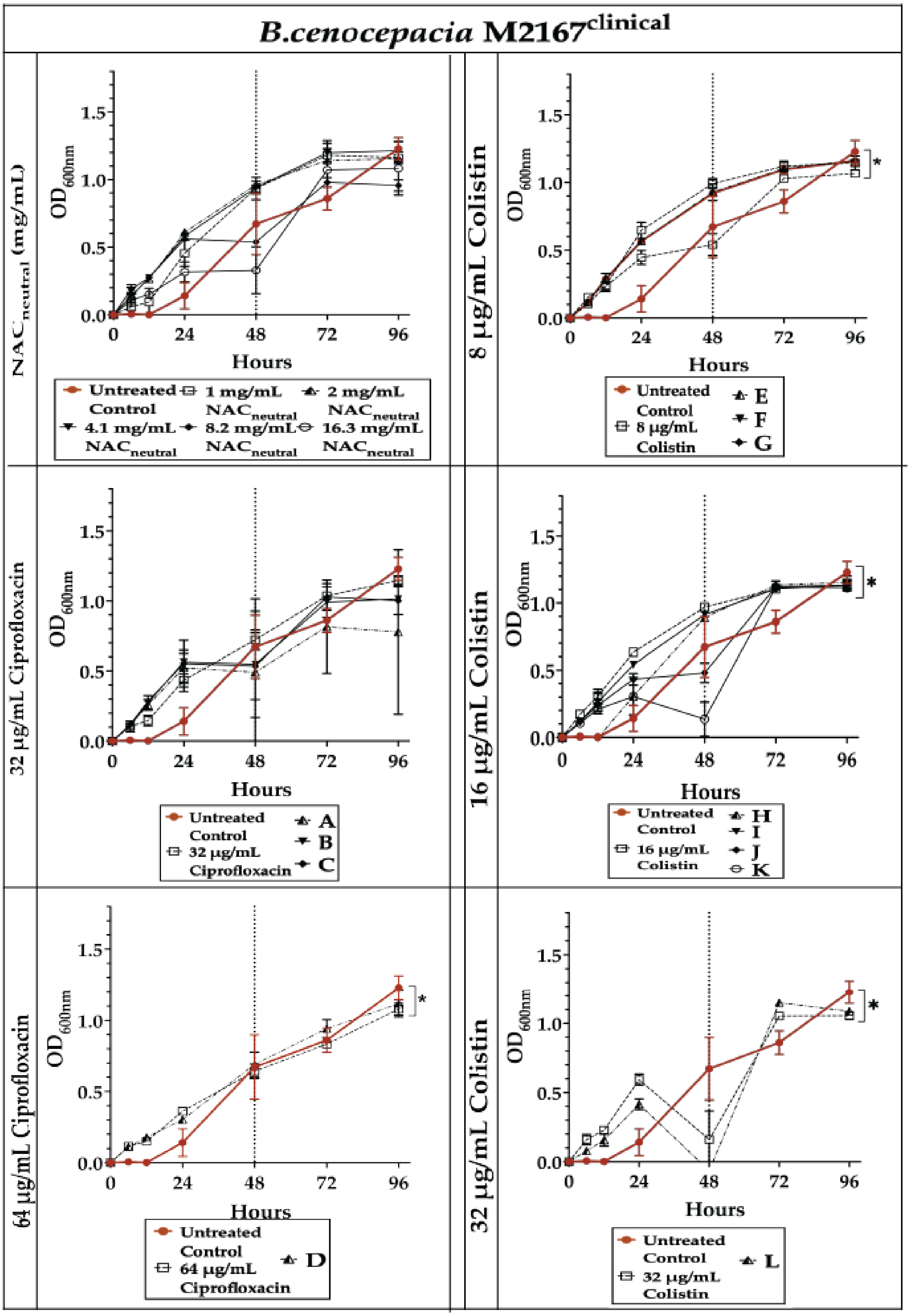
B.cenocepacia M2167clinical
NACneutral (mg/mL)
OD600nm
Hours
Untreated Control
1 mg/mL NACneutral
2 mg/mL NACneutral
4.1 mg/mL NACneutral
8.2 mg/mL NACneutral
16.3 mg/mL NACneutral
8 µg/mL Colistin
Untreated Control
8 µg/mL Colistin
E
F
G
*
32 µg/mL Ciprofloxacin
Untreated Control
32 µg/mL Ciprofloxacin
A
B
C
16 µg/mL Colistin
Untreated Control
16 µg/mL Colistin
H
I
J
K
*
64 µg/mL Ciprofloxacin
Untreated Control
64 µg/mL Ciprofloxacin
D
*
32 µg/mL Colistin
Untreated Control
32 µg/mL Colistin
L
*

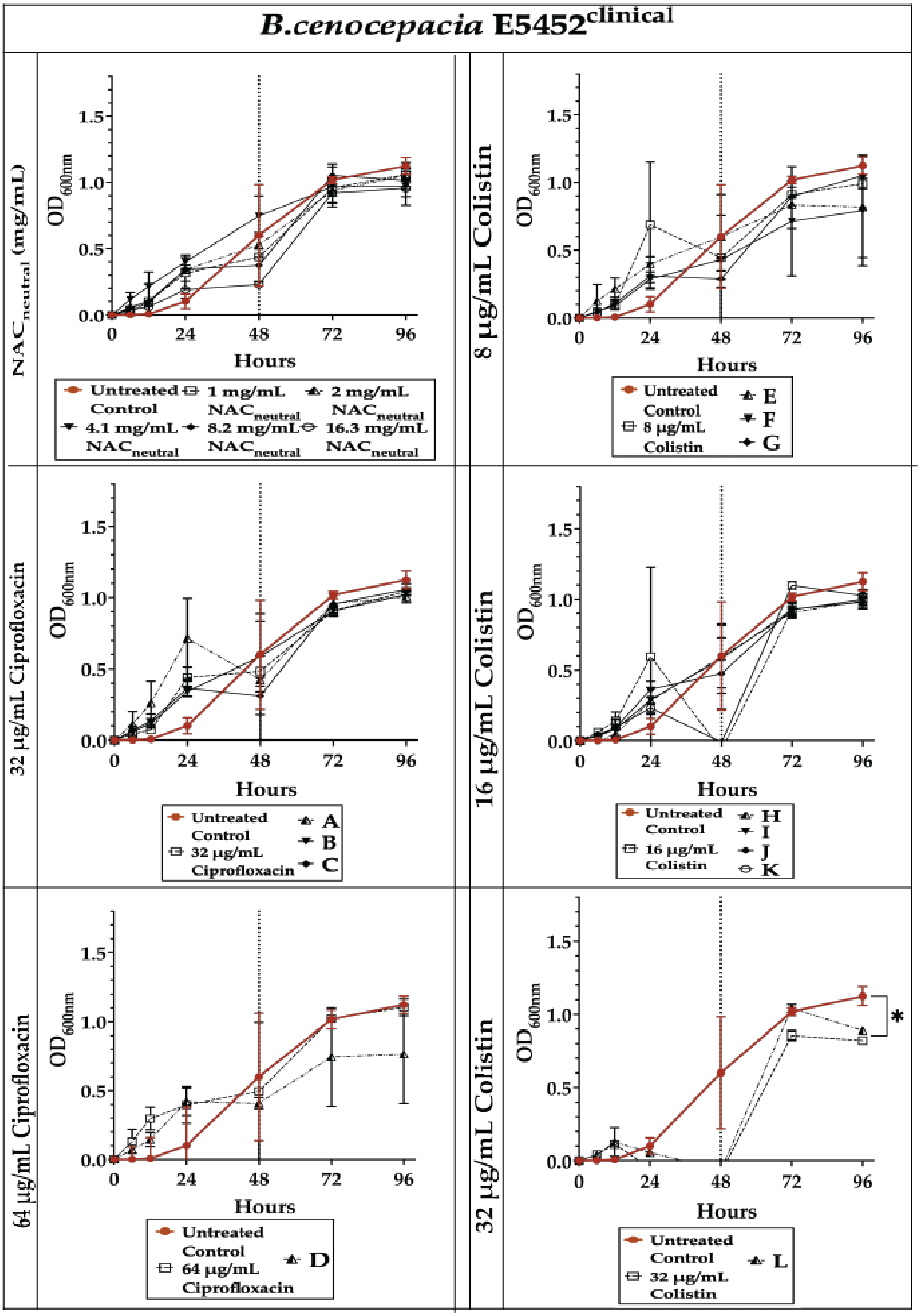
B.cenocepacia E5452clinical
NAC neutral (mg/mL)
OD600nm
Hours
Untreated Control
1 mg/mL NACneutral
2 mg/mL NACneutral
4.1 mg/mL NACneutral
8.2 mg/mL NACneutral
16.3 mg/mL NACneutral
8 µg/mL Colistin
E
F
G
32 µg/mL Ciprofloxacin
A
B
C
16 µg/mL Colistin
H
I
J
K
64 µg/mL Ciprofloxacin
D
32 µg/mL Colistin
L
*

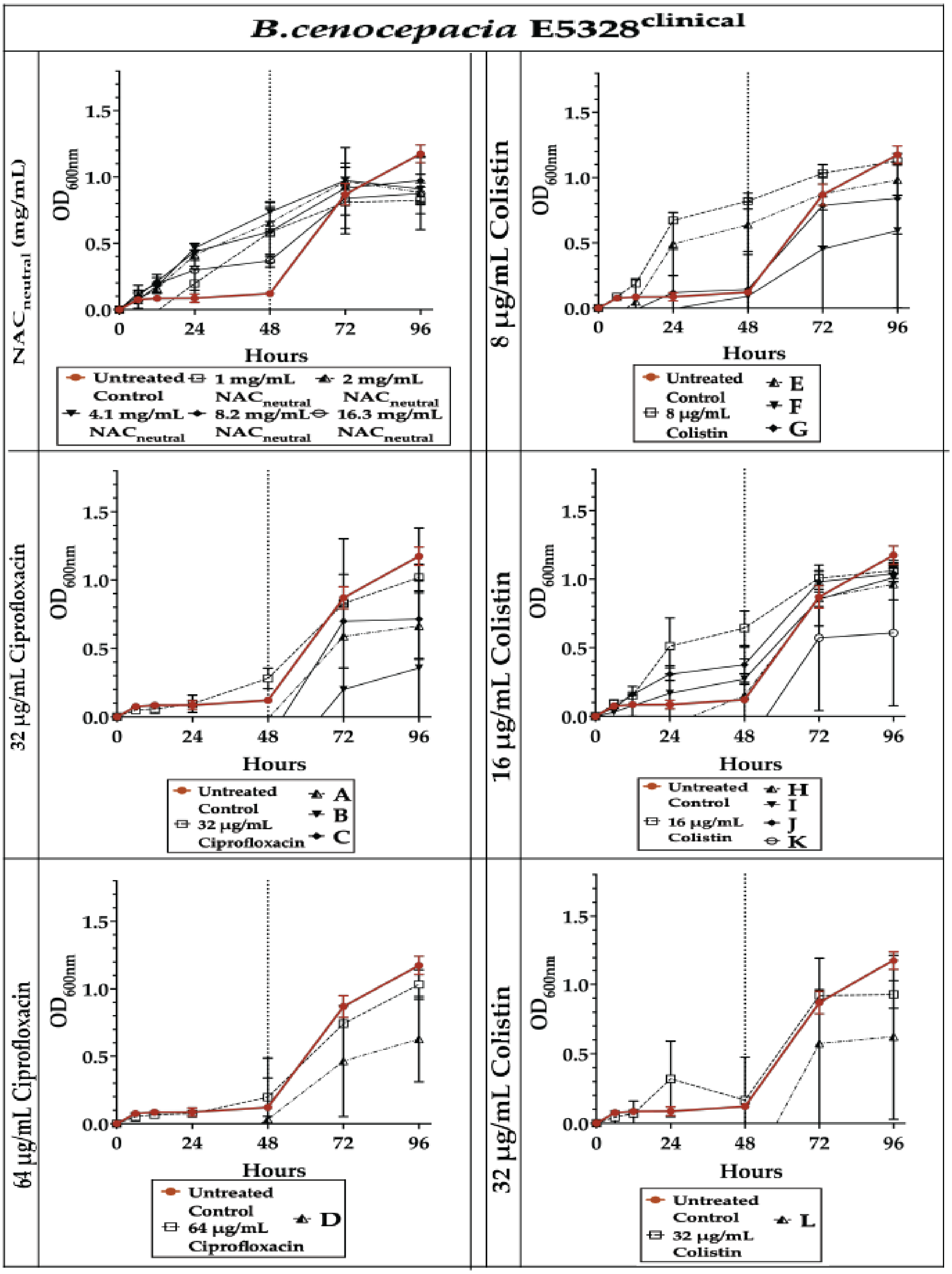

Figure S2. Bacteriostatic effect of NAC$_{neutral}$ and antibiotic combinations on *B. cenocepacia* growth. The vertical dotted line running through the 48 h timepoint signifies the point at which treatment was removed, washed and replaced with fresh TSB. Statistical analyses were performed using unpaired Student's t-tests (with Welch's correction) against final timepoint at 96 h. Significance cut-offs are as follows P ≤ 0.05 (*). Data represent an average of n = 3 biological replicates.

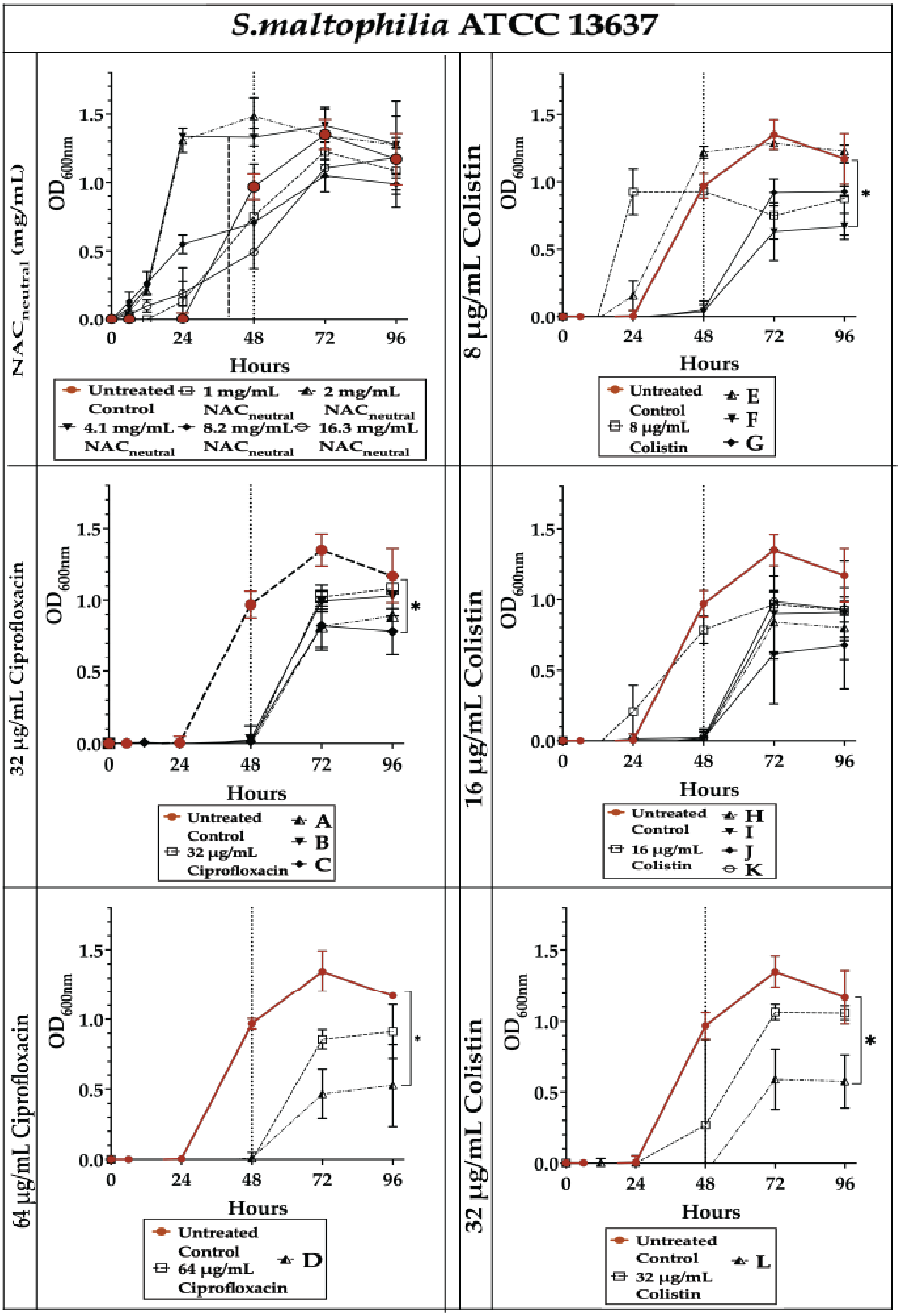
S.maltophilia ATCC 13637
NACneutral (mg/mL)
8 µg/mL Colistin
32 µg/mL Ciprofloxacin
16 µg/mL Colistin
64 µg/mL Ciprofloxacin
32 µg/mL Colistin
OD600nm
Hours
0.0
0.5
1.0
1.5
0
24
48
72
96
*
Untreated Control
1 mg/mL NACneutral
2 mg/mL NACneutral
4.1 mg/mL NACneutral
8.2 mg/mL NACneutral
16.3 mg/mL NACneutral
8 µg/mL Colistin
E
F
G
32 µg/mL Ciprofloxacin
A
B
C
16 µg/mL Colistin
H
I
J
K
64 µg/mL Ciprofloxacin
D
32 µg/mL Colistin
L

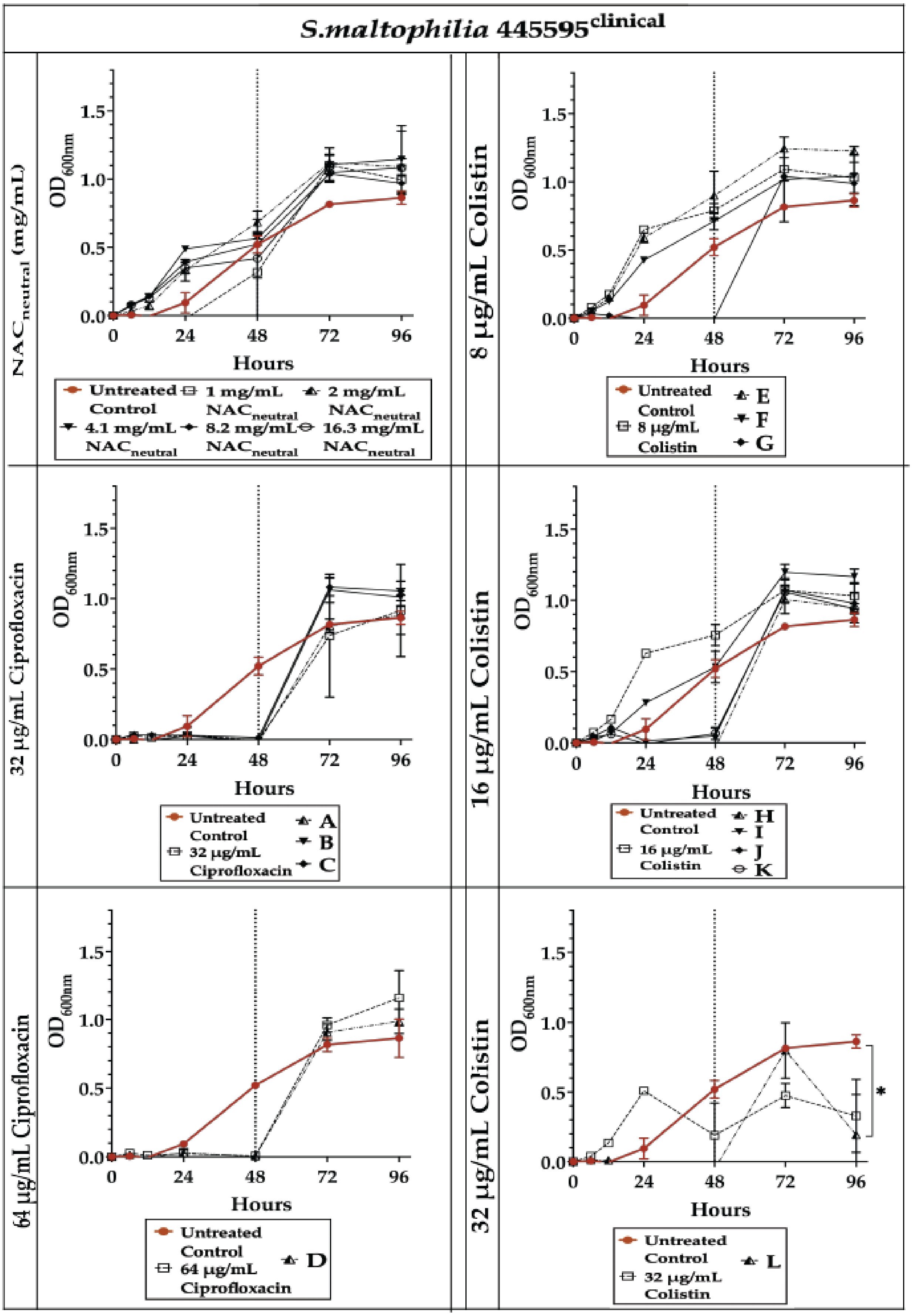
S.maltophilia 445595clinical
NACneutral (mg/mL)
8 μg/mL Colistin
32 μg/mL Ciprofloxacin
16 μg/mL Colistin
64 μg/mL Ciprofloxacin
32 μg/mL Colistin
OD600nm
Hours
Untreated Control
1 mg/mL NACneutral
2 mg/mL NACneutral
4.1 mg/mL NACneutral
8.2 mg/mL NACneutral
16.3 mg/mL NACneutral
8 μg/mL Colistin
E
F
G
32 μg/mL Ciprofloxacin
A
B
C
16 μg/mL Colistin
H
I
J
K
64 μg/mL Ciprofloxacin
D
32 μg/mL Colistin
L
*

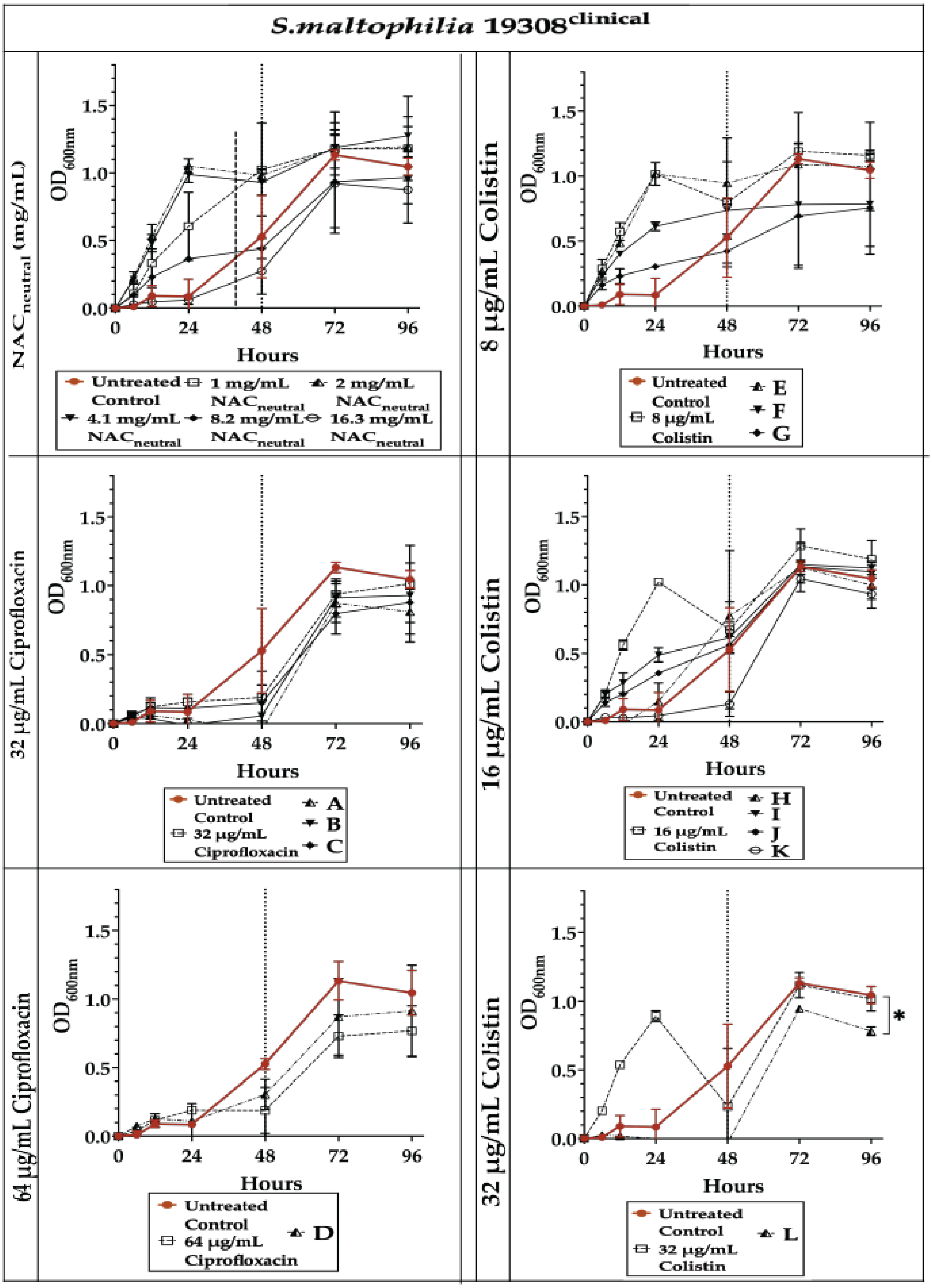

Figure S3. Bacteriostatic effect of $NAC_{neutral}$ and antibiotic combinations on *S. maltophilia* growth. The vertical dotted line running through the 48 h timepoint signifies the point at which treatment was removed, washed and replaced with fresh TSB. Statistical analyses were performed using unpaired Student's t-tests (with Welch's correction) against final timepoint at 96 h. Significance cut-offs are as follows P ≤ 0.05 (*). Data represent an average of n = 3 biological replicates.
